# Treatment of esophageal cancer with radiation therapy: a pan-Chinese survey of radiation oncologists

**DOI:** 10.18632/oncotarget.16858

**Published:** 2017-04-05

**Authors:** Yun Zhang, Jing Liu, Wencheng Zhang, Weiye Deng, Jinbo Yue

**Affiliations:** ^1^ School of Medicine and Life Sciences, University of Jinan Shandong Academy of Medical Sciences, Jinan, Shandong, China; ^2^ Department of Radiation Oncology, Shandong Cancer Hospital and Institute, Shandong Cancer Hospital Affiliated to Shandong University, Jinan, Shandong, China; ^3^ Graduate Education Center, Shandong Academy of Medical Sciences, Jinan, Shandong, China; ^4^ Tianjin Cancer Hospital, Tianjin, China; ^5^ Department of Radiation Oncology, The University of Texas MD Anderson Cancer Center, Houston, Texas, USA; ^6^ Division of Epidemiology, Human Genetics and Environmental Sciences, The University of Texas School of Public Health at Houston, Houston, Texas, USA

**Keywords:** esophageal cancer, radiation therapy, radiation oncologist, questionnaire

## Abstract

Lots of controversies were found about the treatment in relation to radiation therapy (RT) for esophageal squamous cell carcinoma (ESCC). We designed a questionnaire of these controversies to do a pan-Chinese survey of radiation oncologists (ROs). For operable ESCC, 53% ROs chose surgery plus postoperative chemoradiotherapy (CRT), while 40% chose preoperative CRT plus surgery. For target volume of postoperative RT, most ROs (92%) would delineate tumor bed plus involved lymph nodes region before surgery. For definitive RT, most ROs (81%) would give patients higher RT dose to 60–65Gy. For radiation target volume, most ROs would give patients prophylactic irradiation of the bilateral superclavicular-lymph nodes region for cervical ESCC (93%), and the left gastric lymph nodes region for lower thoracic ESCC (72%). For the treatment of mediastinal lymph nodes, 72% ROs preferred elective nodal irradiation, while 28% did the involved nodal irradiation. For concurrent chemotherapy regimen, PF (5-Fu + cisplatin) and TP (cisplatin + paclitaxel) were used widely (49% and 46%, respectively). During simulation, four-dimensional computer tomography (4D CT) was not widely used (48%), even for cervical or lower thoracic ESCC (52%). For daily RT delivery, only 66% ROs would perform imaging guidance RT daily. In summary, more controversies existed in the treatment of ESCC with RT in China, including treatment strategy, radiation dose and target contour. Future goals include standardization of treatment strategy, radiation dose, and target contour, and application of 4D CT and daily imaging guidance, and pursuit of randomized trials in Chinese population.

## INTRODUCTION

Esophageal cancer is the eighth most common cancer and the sixth leading cause of cancer-related mortality in the world [1]. Esophageal cancer has a higher incidence in China than in any other country [2, 3]. The incidence of esophageal cancer was 21.17 per 100000 [3], with the crude mortality rate was 15.58 per 100000, ranking the fourth leading cause of overall cancer deaths in 2012 in China [4].

Radiation therapy (RT) plays an important role in the comprehensive treatment of esophageal cancer [5]. Preoperative chemoradiotherapy (CRT) plus operation has been established as the standard of care for operable patients with esophageal cancer [6], as well as definitive CRT for inoperable patients [7], with high level evidence to improve local control and overall survival (OS) [6, 8].

Although the role of RT is established in the management of esophageal cancer, many controversies exist on the treatment strategy and optimal radiation dose, as a result of the big differences on epidemiology, histology and tumor location for esophageal cancer between different countries [9]. For those reasons, the clinical management of esophageal squamous cell carcinoma (ESCC) including treatment strategy, may be highly variable across different countries and sometimes is likely guided by clinician experiences, even without high level evidence [9, 10]. Involved nodal irradiation (INI) or elective nodal irradiation (ENI) has been hotly debated in recent years [11]. For four-dimensional computer tomography (4D CT), although it is widely used in the simulation for lung cancer [12], how does it extend to esophageal cancer remains unknown in China.

As the highest prevalence country, it's interesting to know how Chinese radiation oncologists (ROs) treat ESCC in daily practice. We aim to perform a national survey of clinical practice among Chinese ROs who treat ESCC. Through the survey, we anticipate finding the controversies on the treatment choice for ESCC, which may promote consensus on the treatment for ESCC in the future as well as trigger ROs’ interests to initiate prospective randomized clinical trials in China.

## RESULTS

### Demographics

A total of 380 ROs, from 27 provinces in China, answered the questionnaire (the survey response rate, 76%). ROs distributed evenly in 27 provinces in China (median, range: 15, 8–26) and 90% ROs came from the first class tertiary hospital. The age and experience of the respondents varied, with 75% ROs more than 40 years old and 59% having treated esophageal cancer more than 10 years. Targeted responses from 224 ROs, older than 40 years and more than ten years experiences in treating esophageal cancer, were finally analyzed.

### Treatment strategy

**Figure 1:**
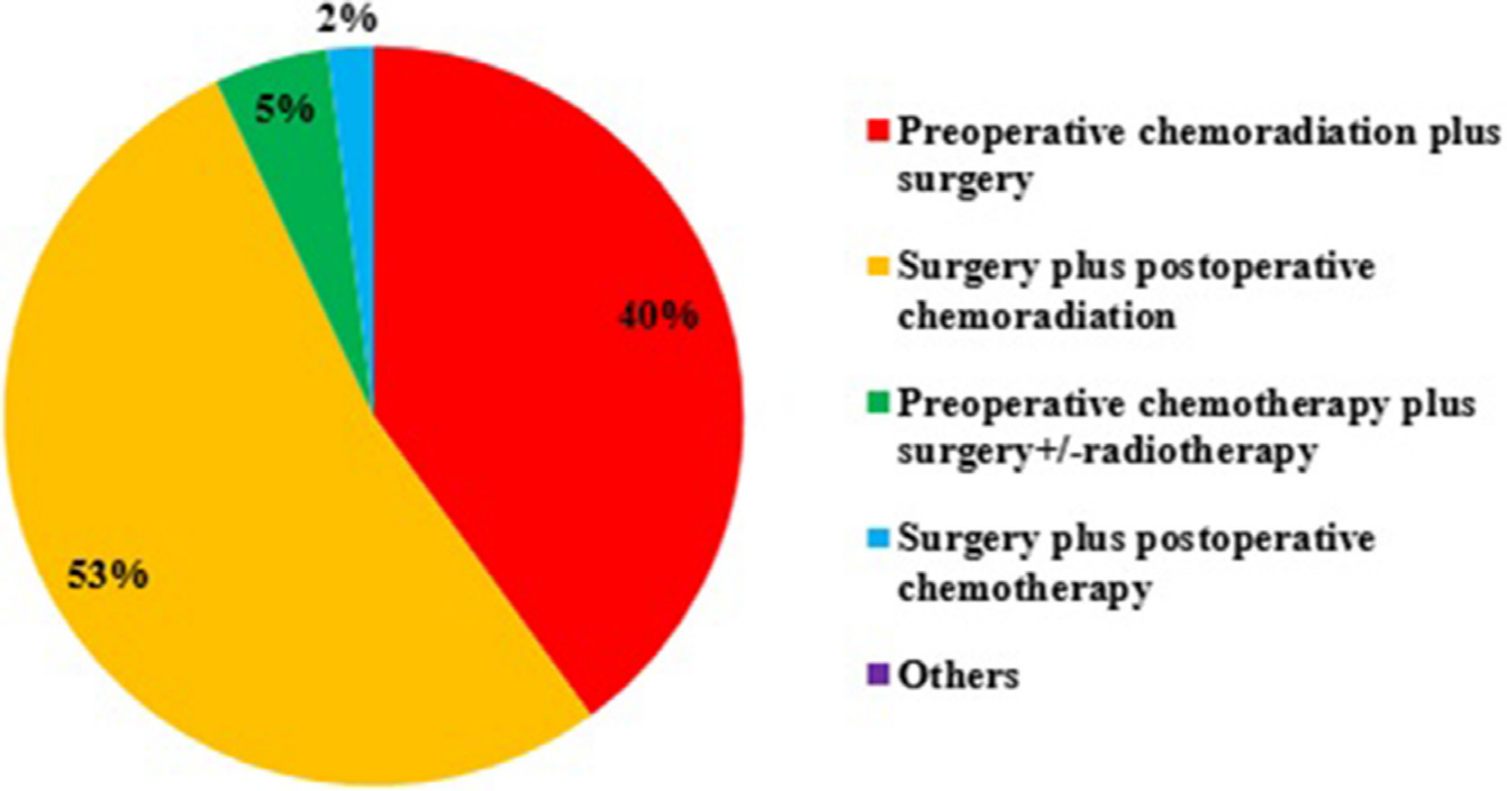
The treatment strategy for operable, locally advanced esophageal squamous cell carcinoma (ESCC)

91% ROs would select challenging cases for multi-disciplinary teamwork (MDT), 7% would discuss all cases, and only 2% never discussed. For operable, locally advanced ESCC, more than half would use surgery plus postoperative CRT rather than preoperative CRT plus surgery (Figure 1). 95% ROs recommended postoperative RT after surgery for locally advanced ESCC. For the reason not using preoperative trimodality, 57% ROs thought the preoperative RT would increase the risk of postoperative complications, such as bleeding, anastomotic leakage and healing delay, although 69% ROs knew the CROSS clinical trial. For the concurrent chemotherapy regimen, 5-Fu plus cisplatin (49%) and weekly paclitaxel plus cisplatin (46%) were commonly used, while the CROSS trial regimen, weekly paclitaxel and carboplatin was not most common (34%).

### Simulation

**Figure 2:**
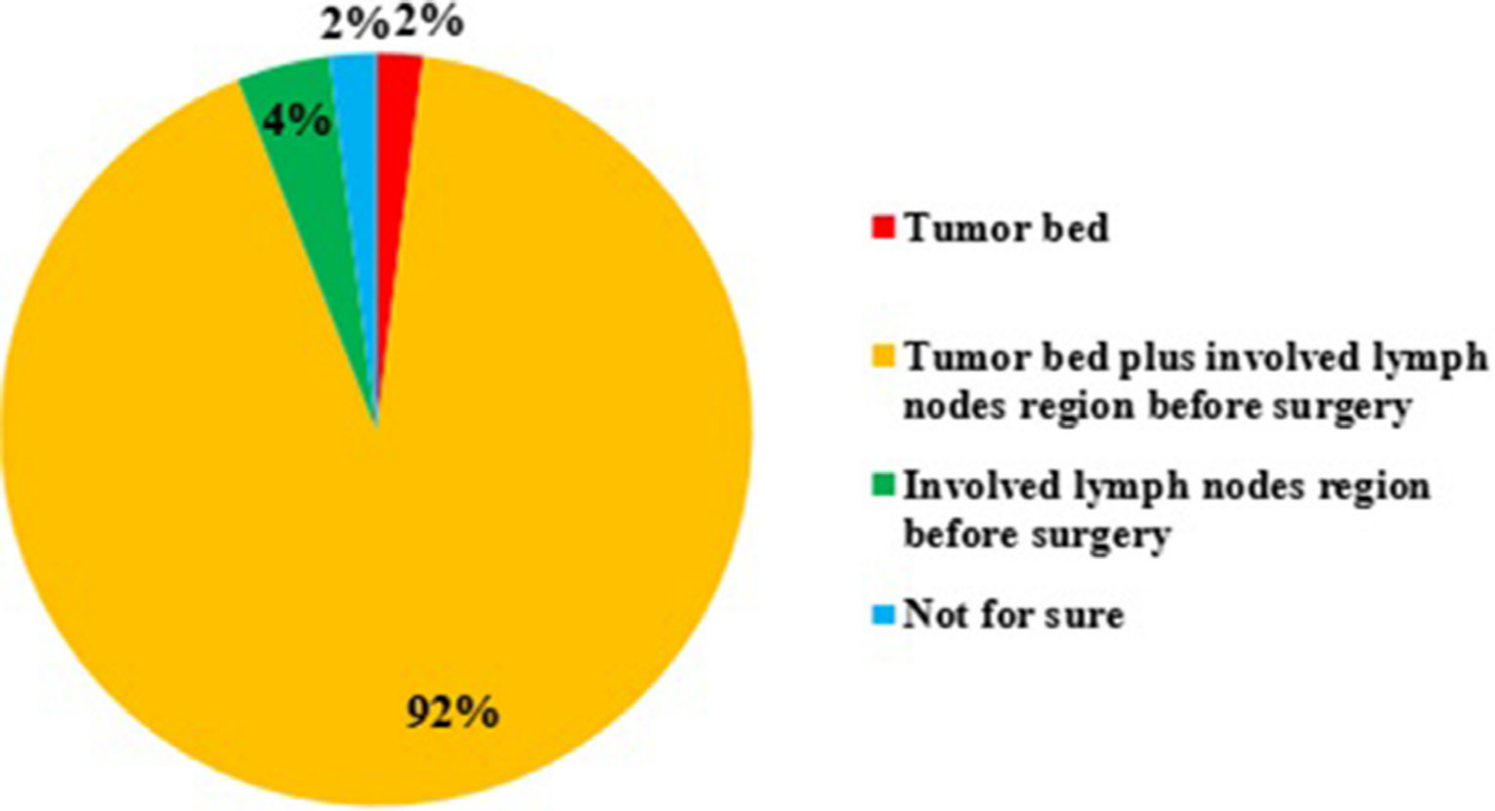
The target volume if giving postoperative radiotherapy for esophageal squamous cell carcinoma (ESCC)

Although 4D CT was available in 89% institutions, 48% ROs, whose institutions have 4D CT, would never use it in routine simulation for esophageal cancer. Only 52% ROs would use 4D CT simulation for cervical or lower thoracic ESCC. In addition to, routine CT simulation, 71% ROs used X-Ray barium fluoroscopy simulation to double check the localization of esophageal tumor. Besides CT, X-Ray barium and endoscopy (55% ROs), positron emission tomography (PET) (53%) or endoscopic mucosal clips (placing at the proximal and distal margins of the tumor, 20%) was used to facilitate tumor contour. After esophagectomy, most ROs would cover tumor bed plus involved lymph node regions before surgery (Figure 2).

### Prescription doses

**Figure 3:**
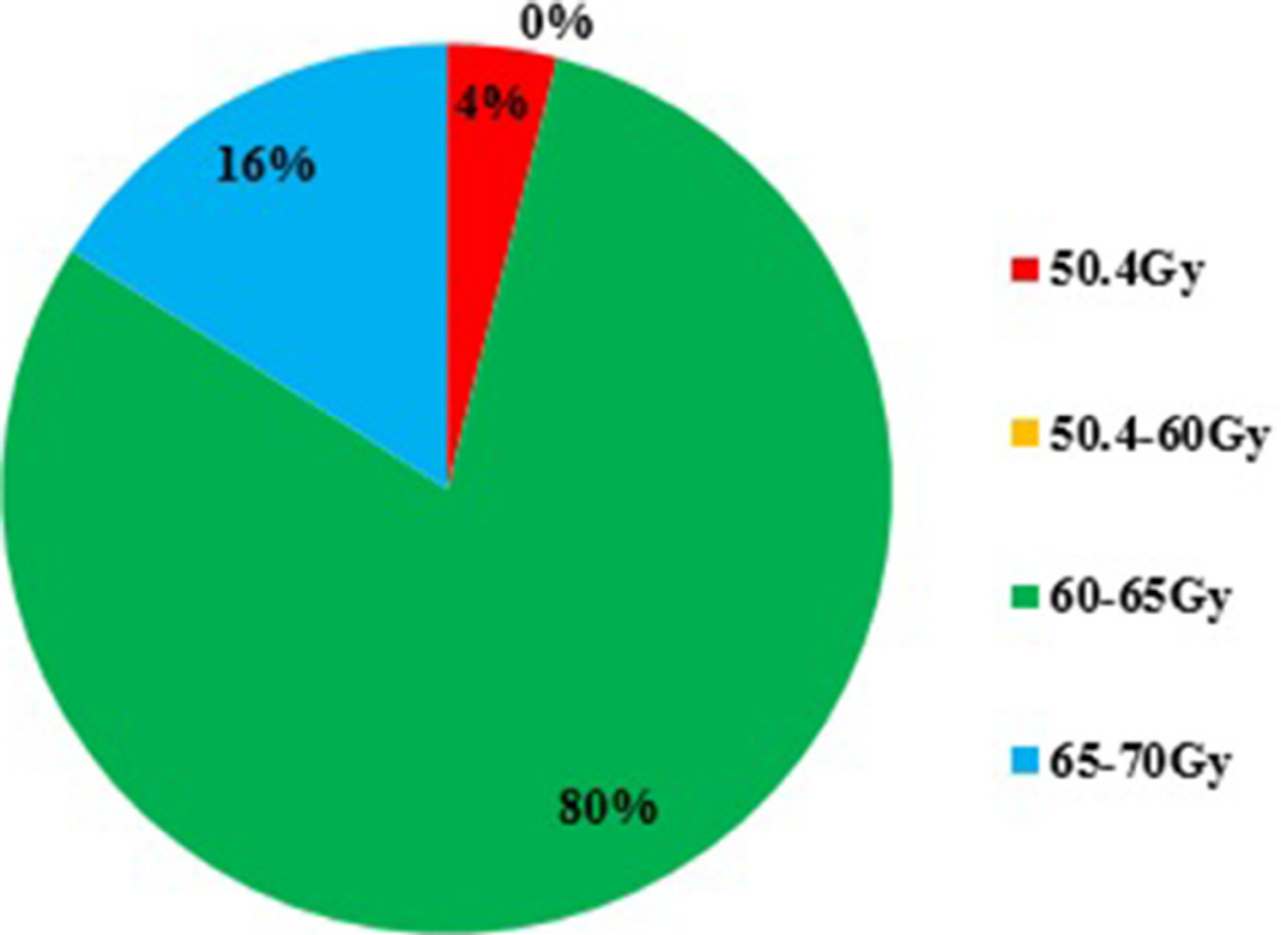
The preferred definitive radiotherapy dose for non-operable, thoracic esophageal squamous cell carcinoma (ESCC)

For inoperable, locally advanced thoracic ESCC, majority of ROs preferred to 60–65Gy rather than 50.4Gy, and minority would perform higher dose to 65–70Gy (Figure 3). The possible reasons why not use 50.4Gy were listed as follows: 51% ROs thought the dose was inadequate according to their experiences; 91% ROs thought there were many differences between Asian and Western countries, including epidemiology, etiology, pathology, and tumor position; 25% ROs thought the recurrence mainly happened in the GTV (gross tumor volume); Some ROs thought the INT-0123 trial used old technical (71%) and bigger margin than that of current clinical practice (75%).

### Target volumes

The clinical target volume (CTV) would be defined as the primary tumor plus 3 cm (75% ROs), 4cm (16%), or 5 cm (9%) expansion superiorly and inferiorly along the length of the esophagus. However, for cervical cancer, only 40% ROs would prefer 3–5 cm expansion superiorly even beyond the upper anatomy bound of esophagus, cricoid cartilage; 32% ROs would define CTV not higher than cricoid cartilage, considering the radiation toxicity of hypopharynx or larynx; 28% ROs would use laryngoscopy to rule out skip esophagus metastasis into hypopharynx or higher, if not, then only expand CTV to cricoid cartilage.

For nodal CTV, the supraclavicular nodes regions were electively treated in patients for cervical (93%) and upper-thoracic (72%) lesions. For lesions of the distal esophagus, the left gastric regions were electively covered for majority of ROs (72%). The left gastric regions would include right cardial nodes (73% ROs), left cardial nodes (64%), nodes along the lesser curvature (67%), nodes along the greater curvature (13%), suprapyloric nodes (9%), infrapyloric nodes (6%), nodes along left gastric artery (49%), nodes along the common hepatic artery (12%), nodes along the celiac axis (20%), nodes at the splenic hilus and artery (4%).

**Figure 4:**
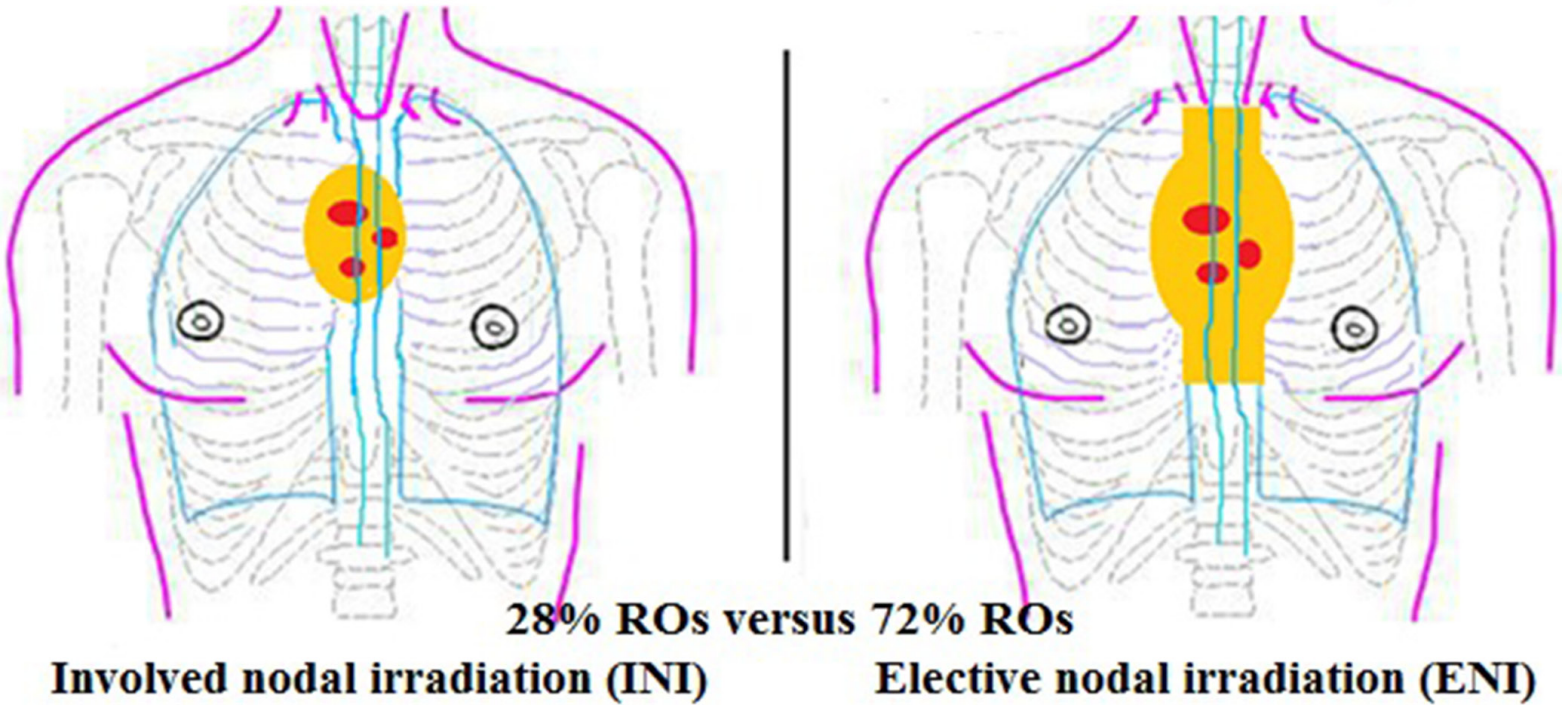
The principal for mediastinal lymph nodes if with positive mediastinal lymph nodes

For the mediastinal nodal CTV (Figure 4), most ROs would prefer ENI, rather than INI. Givng the definitive concurrent CRT, 80% ROs would shrink field during the RT course; the tumor CTV would be coned down to primary tumor plus 1 cm (46% ROs), or 2 cm (34%), or to primary tumor (20%). For organ at risk (OAR), most ROs would outline normal lungs, heart, spinal cord (65%) and normal esophagus (30%); minority would not outline brachial plexus (5%).

### Planning evaluation and RT delivery

68% ROs thought it is not reasonable to use normal tissue dose-volume constraints of non-small cell lung cancer to evaluate OAR for esophageal cancer. During RT, skin tattoo without imaging guided was seldom used to set up patients (13%). Two-dimensional (2D) kilovoltage (kV) orthogonal imaging or electronic portal imaging device (EPID) daily (13%), three-dimensional (3D) kilovoltage (kV) cone-beam computer tomography (CBCT) or megavoltage (MV) CT imaging for Tomotherapy weekly (53%), were used for imaging guided radiotherapy (IGRT).

## DISCUSSION

Results of this pan-Chinese survey of ROs indicated that variation of practice occurs in the treatment of ESCC in China, including treatment strategy, simulation, radiation dose, target contour and RT delivery. Trimodality is one of main treatment strategy for locally advanced ESCC. It's difficult to decide the optimal treatment strategy between neoadjuvant CRT or adjuvant CRT in addition to surgery. There are also active controversies surrounding the dose and the principle of mediastinal lymph nodes irradiation. 4D CT and daily imaging guided RT are necessary to be performed to improve RT accuracy. Future goals include better classification of esophageal cancer, continued pursuit of randomized trials in Chinese population, standardization of treatment strategy, radiation dose, and target contour.

Trimodality strategy of neoadjuvant CRT and surgery have been established as the standard care for locally advanced esophageal cancer [6, 9, 13–15]. However, even majority of ROs (69%) knew the role of the CROSS trial, more than half of ROs chose surgery plus postoperative CRT rather than preoperative CRT plus surgery. What they (57% ROs) concerned is that neoadjuvant CRT may increase the risk of postoperative complications, such as bleeding, anastomotic leakage and healing delay, although CROSS trial has demonstrated that trimodality is associated with similar adverse-event rates compared with surgery alone [15]. The underlying reason is that Chinese ROs believed there is a big difference in treatment strategy between Eastern and Western patients with esophageal cancer, in addition to the clear difference in epidemiology, tumor biology [1]. A new study published in the Nature journal suggested that ESCC is a disease which is completely different from esophageal adenocarcinoma in its molecular characteristics [16], which may change the treatment of ESCC. Current Clinical Practice Guidelines in Oncology (NCCN Guideline) for esophageal cancer are primarily based on the research on Western population. Thus, trimodality may be not suitable for Chinese population [17]. In fact, 23% patients with squamous cell carcinoma were recruited in the CROSS clinical trial and benefited more than those with adenocarcinoma in overall survival (OS) [6]. Besides, the choice might be influenced by the results of a prospective randomized study carried out by Xiao et al. [18], which showed adjuvant radiotherapy could prolong OS. However, a prospective phase III clinical trial from China (NCT01216527), which compared neo-adjuvant CRT followed by surgery for ESCC, had showed promising results that neo-adjuvant CRT improved survival among patients with locally advanced ESCC [19]. The results might change clinical practice for operable ESCC in China in the future. For concurrent chemotherapy regimen, preference was that cisplatin combined 5-Fu or paclitaxel, because Chinese oncologists embraced that cisplatin has more therapeutic efficacy than carboplatin [20]. Besides, part of ROs would choose weekly paclitaxel and cisplatin plus cetuximab, which was based on the EXEL clinical trial (NCT00815308) in China; this trial showed that cetuximab can be safely administered with CRT to patients with locally-advanced ESCC and may improve clinical response rate [21], although RTOG 0436 showed negative results for cetuximab concurrent with RT in esophageal cancer [22].

For non-operative ESCC, RTOG 8501 and INT 0123 clinical trial established definitive CRT using the RT dose of 50.4Gy as the standard of treatment [23, 24]. Recently, a retrospective report based on 6584 patients also has confirmed that dose escalation does not improve OS [23]. However, the optimal RT dose is still hotly debated [9] after the publication of the randomized controlled trial INT-0123 [25], in that 50.4 Gy was used in the North America guideline [26] based on INT-0123 whereas 50–60 Gy was acceptable in the European guideline [27], more than 60 Gy was often used in China and Japan [28–30]. Most Chinese ROs preferred higher dose ≥ 60 Gy in clinical practice in our survey. Although INT-0123 is the only high level based study related with RT dose escalation [31], a recent review paper still commented ‘‘A further dose escalation should be considered as justified” [32]. Radiation dose escalation is a topic of clinical investigation in the setting of esophageal cancer for many years in an attempt to improve outcomes. Chen et al. constructed a propensity score matched study [high dose (60 Gy) vs standard dose (50–50.4 Gy)] based on 648 patients from Taiwan and found that higher dose may lead to better survival for non-operative localized ESCC patients undergoing concurrent chemoradiation (CCRT) [33]. A phase III clinical trial of cancer (NCT01937208) from China is ongoing to compare high dose (60Gy) versus low dose (50 Gy) concurrent with chemotherapy using modern radiotherapy for inoperable ESCC [33]. Meanwhile, in most cases, local failure after combined CRT with a radiation dose of 50.4 Gy for inoperable esophageal cancer develops in the gross tumor volume [30]. A phase I/II clinical trial from M.D. Anderscon cancer center used a simultaneous integrated boost dose of 58.8–63Gy to the gross tumor volume (GTV) concurrent with chemotherapy and showed promising local control [34]. Another dose escalation trial (NCT01843049) from China is ongoing to boost radiation dose within the primary tumor under the guidance of functional imaging for unresectable thoracic esophageal cancer.

With respect to simulation, 4D CT may reduce the motion margin [35, 36] and commonly recommend for cervical or lower thoracic esophageal cancer [37–40]. Although 4D CT is available in 89% institutions, 48% ROs still will not use 4D CT in routine simulation for esophageal cancer. With more clinical studies with 4D CT in China [39, 40], it will be more used in the routine simulation in the future. For target contour, X-Ray barium fluroscopy simulation is commonly used to double check in China. Endoscopic mucosal clips and PET are increasingly applied to define primary esophageal tumor in high-volume hospital in China.

With respect to target volume, our survey indicated that 75% ROs defined CTV margin as the primary tumor plus 3 cm superiorly and inferiorly along the length of the esophagus; this margin was from Gao et al. study in China [41] and consistent with NCCN guideline recommendation [26]. The whole esophagus (RTOG 8501) and 5cm margin (INT 0123) superiorly and inferiorly along the length of esophagus are seldom used in current clinical practice. For cervical cancer, it's more controversial for the CTV margin superiorly along the length of the esophagus. Although it's reasonable that we could use laryngoscopy to rule out skip esophagus metastasis into hypopharynx or higher, if not, then only expand CTV margin superiorly along the length of the esophagus to cricoid cartilage; this method still warranted further clinical trial or recurrence pattern study. 34% ROs would shrink field to 2 cm superiorly and inferiorly plus primary tumor during giving the definitive concurrent CRT as the INT 0123 trial recommended, although it is not evidence based. Further study is warranted to define the optimal CTV used for coning down during RT based on CT or PET imaging matching with the corresponding pathology.

For the optimal target volume of lymph nodes, it is always a controversial topic. It's more consistent that most Chinese ROs would irradiate uninvolved supraclavicular nodes regions for the cervical and the upper thoracic esophageal cancer, the left gastric nodes regions for lower thoracic disease. However, for the mediastinal lymph nodes, ENI versus IFI are hotly debated. Most Chinese ROs would prefer to ENI, because a national randomized, multicenter, phase III clinical trial of CRT for esophageal cancer (NCT00686114) showed that ENI can significantly improve the local control rate and survival rate comparing to IFI [42]. However, several retrospective studies showed IFI is a reasonable treatment strategy with less toxicity as well as not compromising local control and OS [11, 29, 43]. Recently a prospective, multicenter, randomized controlled clinical trial (NCT01551589) conducted in China showed encouraging results for IFI. With INI, the radiation pneumonitis and radiation esophagitis had a marked fall comparing with ENI, while the local regional lymph nodal recurrence rates and distant failure rates were nearly the same between two groups. The almost same OS rates in 1, and 2 years also occurred in two groups [44]. ENI versus INI is needed further verifying which is more reasonable in the future.

On-board imaging is currently a useful tool with great potential to improve awareness of set-up errors and internal motion to ensure that radiation hits the target rather than normal tissue [45, 46]. From the survey, we find that 13% ROs still used skin tattoo to set up patients, and only 13% performed daily KV-EPID or orthogonal imaging. Daily imaging guidance has been demonstrated to accurate the RT dose in target volumes and decrease the toxicity in normal issue [47]. More policy should be instituted by Chinese radiation oncology society to improve widespread adoption of IGRT in clinical practice. In addition, if imaging modalities are available and due to the fact that the dose used for one volume scan is lower than the dose typically used for portal images acquired with the treatment beam, the tested kV CBCT [48] is well suitable for daily position verification in the future .

A limitation of our study is that respondents in the survey might be mostly from large, well-equipped hospitals, which may not represent all ROs in China.

In conclusion, more controversies existed in the treatment of ESCC with RT in China, including treatment strategy, radiation dose and target contour. 4D CT and daily imaging guided RT are necessary to be performed widely used to improve RT accuracy. Future goals include better classification of esophageal cancer, standardization of treatment strategy, radiation dose, and target contour, and application of 4D CT and daily imaging guidance, and pursuit of randomized trials in Chinese population.

## MATERIALS AND METHODS

Five hundred 30-item questionnaires were distributed to Chinese ROs who participated in the first annual meeting of the Chinese Anti-Cancer Association in Jinan, Shandong Province, in August 11–14th, 2016. ROs were first asked details about demographics and clinical experiences, then answered questions pertaining to the clinical management of ESCC with RT. ROs were instructed to select answers closest to their own clinical practice. The questions chiefly covered hot topics on the treatment strategy, RT dose, target delineation, and RT delivery. Supplementary Table 1 showed a complete list of the 30 survey questions. All responses to questions, including partial responses, were deemed eligible for analysis using descriptive statistics.

## SUPPLEMENTARY MATERIALS TABLE





## Abbreviations

RT: radiation therapy
ESCC: esophageal squamous cell carcinoma
ROs: radiation oncologists
CRT: chemoradiotherapy
ENI: elective nodal irradiation
INI: involved nodal irradiation
4D CT: four-dimensional computer tomography
IGRT: image-guided radiotherapy
OS: overall survival
PET: positron emission tomography
GTV: gross tumor volume
CTV: clinical target volume
OAR: organ at risk
2D: two-dimensional
kV: kilovoltage
EPID: electronic portal imaging device
3D: three-dimensional
CBCT: cone-beam computer tomography
MV: megavoltage
CCRT: concurrent chemoradiation
