# Supplementary material for: Treatment of esophageal cancer with radiation therapy: a pan-Chinese survey of radiation oncologists
Source: Oncotarget. 2017 Apr 5;8(21):34946–53. doi: 10.18632/oncotarget.16858 (PMC5471024; doi:10.18632/oncotarget.16858)
Supplement: Supplementary file 1 [file oncotarget-08-34946-s001.pdf]

Treatment of esophageal cancer with radiation therapy: a pan-Chinese survey of radiation oncologists

Supplementary Materials

Supplementary Table 1: Survey questions posed to respondents. See Supplementary_Table_1
