# Supplementary material for: Treatment of esophageal cancer with radiation therapy: a pan-Chinese survey of radiation oncologists
Source: Oncotarget. 2017 Apr 5;8(21):34946–53. doi: 10.18632/oncotarget.16858 (PMC5471024; doi:10.18632/oncotarget.16858)
Supplement: Supplementary file 2 [file oncotarget-08-34946-s002.docx]

| Supplementary Table 1 Survey questions posed to respondents. | |
| --- | --- |
| Q1. | Do you treat esophageal cancer with RT? |
|  | If “No” then please exit the survey. Thank you! |
|  | □ Yes □ No |
| **Demographics** | |
| Q2. | Please inform us of your approximate age. |
|  | □ 25-39 years old □ 40-49 years old □ 50-59 years old □ ≥60 years old |
| Q3. | In which province are you currently working? |
|  | □ Anhui □ Beijing □ Chongqing □ Fujian □ Gansu |
|  | □ Guangdong □ Guangxi □ Guizhou □ Hainan □ Hebei |
|  | □Henan □ Hubei □ Hunan □ Heilongjiang □ Hong Kong |
|  | □ Jiangsu □ Jiangxi □ Jilin □ Liaoning □ Ningxia |
|  | □ Macao □ Neimenggu □ Qinghai □ Shandong □ Shanghai |
|  | □ Shaanxi □ Shanxi □ Sichuan □ Tianjin □ Taiwan |
|  | □ Xinjiang □ Xizang □ Yunnan □ Zhejiang |
| Q4. | How many years have you treated esophageal cancer? |
|  | □ <5 years □ 10 - 5 years □ 10-15 years □ 15-20 years □ > 20 years |
|  | **Treatment strategy** |
| Q5. | How often do you review cases of esophageal cancer in a multidisciplinary case conference? |
|  | □ All of the new cases were discussed |
|  | □ Only challenging cases were selected |
|  | □ Never discussed |
| Q6. | What`s your treatment strategy for operable, locally advanced ESCC (T≥T_3_ or lymph nodes positive)? |
|  | □ Preoperative CRT plus surgery |
|  | □ Surgery plus postoperative CRT |
|  | □ Preoperative chemotherapy plus surgery+/-radiotherapy |
|  | □ Surgery plus postoperative chemotherapy |
|  | □ Others |
| Q7. | Which is the following standard treatment established by CROSS clinical trial in your mind? |
|  | □ Definitive concurrent CRT |
|  | □ Neoadjuvant chemotherapy plus surgery |
|  | □ RT alone |
| Q8. | Do you think neoadjuvant CRT would increase the complication after esophagectomy? Such as bleeding, anastomotic leakage and healing delay? |
|  | □ Yes □ No |
| Q9. | Which do you think is not appropriate for esophagectomy? |
|  | □ Cervical esophageal cancer |
|  | □ Upper thoracic esophageal cancer |
|  | □ Middle thoracic esophageal cancer |
|  | □ Lower thoracic esophageal cancer |
| Q10. | What`s your favorite concurrent chemotherapy regime for esophageal squamous carcinoma? Please select all that apply. |
|  | □ Weekly paclitaxel and carboplatin (CROSS clinical trial regimen) |
|  | □ Weekly paclitaxel and cisplatin |
|  | □ Weekly paclitaxel and cisplatin plus cetuximab synchronization |
|  | □ 5-Fu and cisplatin, 28 days one cycle, 4 cycles (RTOG 8501 regimen) |
|  | □ Docetaxel and cisplatin, 21 days one cycle |
|  | □ Weekly docetaxel and 5-Fu |
|  | □ Xeloda® (capecitabine) |
|  | □ Others |
| **Simulation** | |
| Q11. | Is 4D CT available in your institution? |
|  | □ Yes □ No |
| Q12. | Would you use 4D CT for esophageal tumor if your institution have 4D CT? |
|  | □ Never used |
|  | □ Only would use it for cervical or lower thoracic ESCC |
| Q13. | Would you use X-Ray barium fluoroscopy simulation to double check the localization of esophageal tumor? |
|  | □ Yes □ No |
| Q14. | Which method would you use to facilitate tumor contour? Please select all that apply. |
|  | □ CT, X-Ray barium and endoscopy |
|  | □ PET/CT |
|  | □ Endoscopic mucosal clips (placing at the proximal and distal margins of the tumor) |
|  | □ Others |
| **Prescription dose** | |
| Q15. | What is your preferred definitive RT dose for inoperable, thoracic ESCC? |
|  | □ 50.4Gy □ 50.4-60Gy □ 60-65Gy □ 65-70Gy |
| Q16. | What is the preferred definitive RT dose for cervical ESCC? |
|  | □ 50.4Gy □ 50.4-60Gy □ 60-65Gy □ 65-70Gy |
| Q17. | If you didn’t use 50.4Gy as INT 0123 established for definitive RT dose, what are the possible reasons? Please select all that apply. |
|  | □ It is inadequate dose for esophageal squamous carcinoma by experience. |
|  | □ There are many differences between Asian and Western countries, including epidemiology, etiology, pathology, and tumor position. |
|  | □ In most cases, higher dose may bring better local control because local failure after combined CRT with a radiation dose of 50.4 Gy for unrespectable esophageal cancer develops in the GTV. |
|  | □ The INT-0123 trial is performed from 1995 to 1999, and since then, notable technical advances in radiation treatment planning and delivery have occurred |
|  | □ The margins for both the primary and high-dose volumes in INT-0123 triala are significantly larger than those used in current clinical practice, resulting in higher dose to the normal esophagus, heart, and lungs, which would have increased the possibility of toxicity. |
| **Target volumes** | |
| Q18. | What’s the expansion superiorly and inferiorly along the length of esophagus to the primary tumor when you define the CTV? |
|  | □ 3cm □ 4cm □ 5cm □ All the esophagus |
| Q19. | What’s the expansion superiorly along the length of esophagus to the primary tumor when you define the CTV for cervical ESCC? |
|  | □ 3-5cm expansion superiorly even beyond the upper anatomy bound of esophagus cricoid cartilage. |
|  | □ Not higher than cricoid cartilage, considering the radiation toxicity of hypopharynx or larynx |
|  | □ First, use laryngoscopy to rule out skip esophagus metastasis into hypopharynx or higher, if not, then only expand CTV to cricoid cartilage. |
| Q20. | Would you shrink field during the course of RT, when you give the definitive concurrent CRT? |
|  | □ Yes □ No |
| Q21. | What’s the expansion superiorly and inferiorly along the length of coning down to the primary tumor when you shrink the field during RT? |
|  | □ Cone down to 1cm plus primary tumor |
|  | □ Cone down to 2cm plus primary tumor |
|  | □ Cone down to primary tumor.  Resp. 10  Reviewer #3 |
| Q22. | What’s your target volume if giving postoperative RT for ESCC (T≥T_3_ or lymph nodes positive)? |
|  | □ Tumor bed |
|  | □ Tumor bed plus involved lymph nodes region before surgery |
|  | □ Involved lymph nodes region before surgery |
|  | □ Not for sure |
| Q23. | Would you electively irradiate bilateral supraclavicular lymph nodes region for the cervical ESCC? |
|  | □ Yes □ No |
| Q24. | Would you electively irradiate bilateral supraclavicular lymph nodes region for the upper thoracic ESCC? |
|  | □ Yes □ No |
| Q25. | Would you electively irradiate the left gastric lymph nodes region for the lower thoracic ESCC? |
|  | □ Yes □ No |
| Q26. | Which lymph node regions you would include for the left gastric lymph nodes region as elective target volume? Please select all that apply. |
|  | 1. Right cardial nodes |
|  | 2. Left cardial nodes |
|  | 3. Nodes along the lesser curvature |
|  | 4. Nodes along the greater curvature |
|  | 5. Suprapyloric nodes |
|  | 6. Infrapyloric nodes |
|  | 7. Nodes along left gastric artery |
|  | 8. Nodes along the common hepatic artery |
|  | 9. Nodes along the celiac axis |
|  | 10. Nodes at the splenic hilus and artery |
| Q27. | What`s your principal for mediastinal lymph nodes if with positive mediastinal lymph nodes? |
|  | □INI |
|  | □ENI, such as, if with 2R positive lymph nodes, the 2, 4 lymph nodes stations would be covered in the target volume. |
| Q28. | Would you routinely outline the following organs and give evaluation for endangering organ? Please select all that apply. |
|  | □ Normal lungs, heart, spinal cord |
|  | □ Normal esophagus |
|  | □Brachial plexus ,especially for cervical or upper thoracic ESCC |
| **Planning evaluation and RT delivery** | |
| Q29. | Do you think it is reasonable to use normal tissue dose-volume constraints of non-small cell to evaluate OAR for esophageal cancer? |
|  | □ Yes □ No |
| Q30. | Which method would you use to set up patients daily? |
|  | □ Depending on skin tattoo only |
|  | □ Depending on skeleton matched by EPID or KV-orthogonal imaging daily |
|  | □Depending on tumor matched by 3D KV-CBCT or MV-CT daily |
|  | □Others |

[Abbreviations：RT, radiation therapy; ESCC, esophageal squamous cell carcinoma; CRT, chemoradiotherapy; 4D CT, four-dimensional computer tomography; PET, positron emission tomography; GTV, gross tumor volume; CTV, clinical target volume; INI, involved nodal irradiation; ENI, elective nodal irradiation; OAR, organ at risk; EPID, electronic portal imaging device; kV, kilovoltage; 3D, three-dimensional; CBCT, cone-beam computer tomography; MV megavoltage](http://xueshu.baidu.com/s?wd=paperuri%3A%281f2239c442ba36582b7c44ce510163c6%29&filter=sc_long_sign&tn=SE_xueshusource_2kduw22v&sc_vurl=http%3A%2F%2Fwww.ncbi.nlm.nih.gov%2Fpubmed%2F8587942&ie=utf-8&sc_us=10942901053318972267)
